# Effect of spirotetramat and fluensulfone on population densities of *Mesocriconema xenoplax* and *Meloidogyne incognita* on peach

**DOI:** 10.21307/jofnem-2019-012

**Published:** 2019-04-17

**Authors:** Andrew M. Shirley, James P. Noe, Andrew P. Nyczepir, Phillip M. Brannen, Benjamin J. Shirley, Ganpati B. Jagdale

**Affiliations:** 1 Department of Plant Pathology, University of Georgia, Athens, GA, 30602; 2 USDA-ARS, SE Fruit and Tree Nut Research Lab, 21 Dunbar Rd, Byron, GA, 31008

**Keywords:** Fluensulfone, Management, *Meloidogyne incognita*, Mesocriconema xenoplax, Nematicide, Peach tree decline, Peach tree short life, Ring nematode, Root-knot nematode, Spirotetramat

## Abstract

Management of plant-parasitic nematodes (PPNs) on peach is needed for a longer period of time than is typically afforded by pre-plant fumigant nematicides. Two post-plant nematicides, spirotetramat and fluensulfone, were evaluated for control of *Meloidogyne incognita* and *Mesocriconema xenoplax* under laboratory and greenhouse conditions. In vitro assays were conducted to test the effect of spirotetramat at 0.017 and 0.026 kg a.i./ha and fluensulfone at 3.92 kg a.i./ha on the mobility of both *M. incognita* and *M*. *xenoplax* in 24-well plates for 24, 48, and 72 hr, compared to a water control. Both fluensulfone and spirotetramat reduced mobility of *M. xenoplax*, but only fluensulfone reduced the mobility of *M. incognita*, compared to the untreated control. In peach greenhouse trials, both spirotetramat at 0.017 kg a.i./ha and fluensulfone at 3.92 kg a.i./ha reduced *M. incognita* numbers by 62 and 77% at 40 d after inoculation (DAI), respectively; neither chemical reduced populations at 70 DAI. Fluensulfone reduced *M. xenoplax* numbers by 84, 94, and 96% at 30, 60, and 90 DAI, respectively. No effects were observed for spirotetramat on *M. xenoplax*. At 40 DAI, dual applications of spirotetramat 30 d apart reduced *M. incognita* numbers by 58 and 54% for both 0.017 and 0.026 kg a.i./ha rates, respectively; no reductions were observed at 70 DAI. No effect was seen for a dual application of spirotetramat on *M. xenoplax*. These post-plant nematicides may provide additional options for management of PPNs on peach.

In the State of Georgia, peach production is a $42.1 million industry with production ranking third behind California ($350 million) and South Carolina ($67.9 million) (USDA Georgia Agricultural Facts, 2017). Nematode-related diseases pose severe production constraints on peach in the southeastern United States. The ring nematode, *Mesocriconema xenoplax* (Raski) Loof & de Grisse [= *C. xenoplax* (Raski) Luc and Raski], is arguably one of the most important nematode pathogens on peach [*Prunus persica* (L.) Batch] due to its association with the disease complex known as peach tree short life (PTSL) (Brittain and Miller, 1978; Nyczepir et al., 1983; Nyczepir, 1989). In a survey of commercial peach orchards in South Carolina and Georgia, *M. xenoplax* was detected in 100% of soil samples collected from those orchards where PTSL was present (Nyczepir et al., 1985). Peach tree decline, unlike PTSL, is often associated with the root-knot nematode (*Meloidogyne* spp.) and the root-lesion nematode (*Pratylenchus vulnus*) (Ritchie and Clayton, 1981; Nyczepir, 2011b). The root-knot nematodes (RKNs), *M. incognita* and *M. javanica*, were found in 95 and 5% of peach orchards surveyed in South Carolina, respectively (Nyczepir et al., 1997). Aboveground symptoms associated with RKN feeding include stunted plant growth, promotion of early defoliation in severely stunted plants, and a severe reduction in fruit yields. Belowground symptoms include reduced, malformed, and severely galled root systems. High RKN infestation could lead to peach tree death (Nyczepir et al., 1993).

Currently, a pre-plant fumigation with 1-3 dichloropropene (Telone II, DowAgrosciences) combined with the use of a resistant rootstock is recommended for the management of nematodes in peach orchards (Nyczepir, 1991; Beckman and Nyczepir, 2011). In addition, pre-plant crop rotations with bahia and tall fescue grasses and wheat have been recommended for the suppression of peach nematodes in the Southeast (Nyczepir and Meyer, 2010; Nyczepir, 2011a; Meyer et al., 2013). These practices are initially successful in suppressing nematode populations, but after the first two or three years, the nematode populations recover to damaging levels. This can threaten the productivity and life of an orchard, making it susceptible to secondary disorders like PTSL, peach tree decline, and nepoviruses (Brittain and Miller, 1978; Ritchie and Clayton, 1981; Beckman and Nyczepir, 2011; Nyczepir, 2011b). In 1992, it was estimated that over $6 million is lost each year in South Carolina to PTSL alone (Miller, 1994). Therefore, there is a need for better management practices for the control of peach nematodes. Given the rising cost of pre-plant fumigant applications and the short-lived effects of nematode control by fumigants, there is also a need for the development of sustainable post-plant nematode control strategies in perennial crops including peach (McKenry et al., 2009, 2010, 2011).

Spirotetramat (Movento™, Bayer CropScience) is currently marketed as a safe broad-spectrum systemic insecticide and nematicide with a very low level of mammalian toxicity (>5,000 mg a.i./kg bw), used to control insects in multiple crops and nematodes in stone fruit and tree nuts. When sprayed on the leaf surface, spirotetramat hydrolyzes to its -enol form in the leaf tissue and then it is translocated through the phloem and xylem to both leaf and root apical meristems. Spirotetramat is a Group 23 lipid biosynthesis inhibitor; it acts on and reduces egg laying capacity (fecundity) and viability of eggs (fertility), and affects a process of ecdysis (leading to the incomplete shedding of the cuticle during molting) when ingested by organisms such as aphids (Bruck et al., 2009). The residual activity of spirotetramat in the soil is very short-lived with around a 90% reduction in 1 to 4 d; however, it maintains high toxic levels for more than 2 wk within plants (Bruck et al., 2009). McKenry et al. (2009) applied spirotetramat at <100 ml/ha to *Vitis* spp., *Citrus* spp., and *Juglans* spp. and observed that the populations of *Xiphinema* spp. and *M. xenoplax* were reduced 36 and 56 d after treatment, respectively. It was also observed that 50% of the population of all plant-parasitic nematodes, including *Meloidogyne* spp., were reduced if irrigation was withheld for up to 2 wk (McKenry et al., 2009). Smiley et al. (2011) applied spirotetramat at 0.088 kg a.i./ha to wheat fields that were infested with the cyst nematode, *Heterodera avenae*, and found that spirotetramat reduced population densities of *H. avenae* by 78%.

Fluensulfone (Nimitz™, ADAMA Agricultural Solutions Ltd., Raleigh, NC) is not currently labeled as a nematicide for use against peach nematodes, but it may be a promising post-plant nematicide for use on peach, as it has exhibited strong nematicidal activity against nematodes in other cropping systems. Fluensulfone belongs to the fluoroalkenyl group, has low mammalian toxicity (between 500 and 1,000 mg/kg), and is non-toxic to honey bees and birds (Everich and Schiller, 2009). Fluensulfone is generally applied through drip irrigation or drench application for nematode control. Trials conducted by Oka et al. (2009) on the efficacy of fluensulfone against *M. javanica* on tomato demonstrated that a drench application of fluensulfone at rates of 0.5, 1.0, 2.0, and 4.0 mg a.i./L indicated that all rates significantly reduced galling and eggs counts compared to the control. In another trial, fluensulfone was applied as a pre-plant application at 2.1, 4.2, 6.3, and 8.3 L/ha, and a pre-plant and post-plant application at 8.3 and 4.2 L/ha, respectively. All of the rates of fluensulfone, except for the pre-plant application of 4.2 L/ha, had significantly lower gall ratings compared to the control (Driver and Louws, 2010). Although Morris et al. (2016) applied fluensulfone at 3 kg a.i./ha either via a pre-plant incorporation or drip irrigation against *Meloidogyne* spp. in vegetable fields, they only observed reduced galling on the roots when fluensulfone was applied through the drip.

Since both spirotetramat and fluensulfone nematicides have shown detrimental effects on plant-parasitic nematodes of different crops, we hypothesized that the applications of these nematicides as post-plant treatments would be effective in controlling nematodes on peach. Therefore, the first objective of this research was to study the effect of both spirotetramat and fluensulfone on the mobility of *M. incognita* and *M. xenoplax* in an in vitro assay. The second objective was to evaluate their effect on population densities of both *M. incognita* and *M. xenoplax* on peach under greenhouse conditions.

## Materials and methods

### Sources of nematodes and inoculum

Populations of *M. incognita* and *M. xenoplax* were originally isolated from peach orchards in Georgia and maintained on eggplant (*Solanum melongena* cv. ‘Black Beauty’) and peach (*Prunus persica* cv. ‘Lovell’ and ‘Nemaguard’), respectively, in a greenhouse. Mixed stages of *M. xenoplax* and eggs of *M. incognita* were collected from the culture medium and eggplant roots using the centrifugal-flotation method (Jenkins, 1964) and a 0.5% NaOCl solution (Hussey and Barker, 1973), respectively, and used as inoculum in both in vitro assays and greenhouse trials.

### Sources of rootstocks

The seedlings of peach rootstocks, including ‘Lovell’ and ‘Nemaguard’ that are susceptible to *M. incognita* and *M. xenoplax*, respectively, were grown from seed provided by the Southeastern Fruit and Tree Nut Research Laboratory of the USDA-ARS. Briefly, the seeds of both rootstocks were germinated and planted into 28 × 56 × 6 cm planting trays containing 9.4 L of pasteurized Fafard germinating mix (Fafard, Sun Gro Horticulture), and allowed to grow for 4 wk in a greenhouse at 27 ± 5 °C until used in the trials.

### Sources of nematicides

Commercial formulations of spirotetramat (Movento™) and fluensulfone (Nimitz™) were obtained from Bayer CropScience (Bayer AG, Rhine, Germany) and ADAMA (Agricultural Solutions Ltd., Raleigh, NC), respectively. Their efficacies were evaluated against *M. incognita* and *M. xenoplax* in both in vitro assays and greenhouse trials during 2011 to 2012. Spirotetramat and fluensulfone were tested in a single trial against *M. xenoplax*, but two trials were conducted for the evaluation of spirotetramat and fluensulfone against *M. incognita*. Initially, fluensulfone and spirotetramat were both evaluated for efficacy through foliar applications against *M. incognita*, but the foliar application of fluensulfone lead to phytotoxicity among all plants treated (data not shown). Thus, a separate *M. incognita* trial was conducted with fluensulfone alone as a soil drench application, where no phytotoxicity was observed (phytotoxic data not shown). Therefore, fluensulfone was applied as soil drench and spirotetramat was applied as foliar application against both *M. incognita* and *M. xenoplax* nematodes in all the trials presented here.

### In vitro assays

An in vitro assay was conducted in 24-well plates to evaluate the efficacy of spirotetramat and fluensulfone against *M*. *incognita* second-stage juveniles (J2) and *M. xenoplax* at room temperature (25 ± 2°C). This assay was comprised of four treatments, including two rates of spirotetramat at 0.017 and 0.026 kg a.i./ha, one rate of fluensulfone at 3.92 kg a.i./ha, and a control (sterile tap water). There were six replications per treatment and the assay was repeated once. Rates were determined from the most efficacious rates used in previously published trials (McKenry et al., 2009, 2010, 2011; Csinos et al., 2010). For spirotetramat treatments, 92 and 140 µl of spirotetramat (0.017 and 0.026 kg a.i./ha, respectively) were mixed with 50 ml of sterile water. For fluensulfone, 1.67 µl of fluensulfone (3.92 kg a.i./ha) was mixed with 100 ml of sterile water. From each prepared stock solution, 1 ml of each concentration was placed in each well and a 1 ml suspension of approximately 1,000 *M. xenoplax* mix stages were added to each well. For the *M. incognita* trials, a 1 ml suspension of 500 J2 was prepared for the first in vitro assay and 1,000 J2 were used for the repeated in vitro assay. The percentage of nematode mortality was determined 24, 48, and 72 hr after initial exposure to the treatments. To determine nematode mortality, a 500 μl of thoroughly mixed sub-sample from each well, containing on average 105 *M. xenoplax* nematodes and 130 *M. incognita* juveniles, was added to 5-cm-diameter glass dishes containing 3 ml of sterile water, and allowed to diffuse into the solution for 1 hr. The percentage of nematode mortality was determined by counting the numbers of all mobile and non-mobile nematodes under a stereomicroscope. The non-mobile nematodes were considered alive if there was a response to probing with a fine probe. Each bioassay was repeated once for each nematode species and data combined for analysis.

### Effect of a single application of spirotetramat and fluensulfone on population density of *M. incognita*

Due to phytotoxicity of fluensulfone to ‘Lovell’ rootstock when foliarly applied, a spirotetramat foliar treatment was evaluated in a separate trial from a soil application of fluensulfone, under greenhouse conditions during 2011. For both *M. incognita* trials, four-weeks old seedlings of Lovell rootstock were transplanted into 20-cm-diameter standard clay pots containing 3.4 L of pasteurized loamy sand soil mixture of 25% field soil, 50% sand, and 25% Fafard germinating mix (Fafard, Sun Gro Horticulture), and allowed to establish for two weeks in a greenhouse at 27 ± 5 °C. Two weeks after transplanting, each seedling was inoculated with 20,000 *M. incognita* eggs/pot. Then, 10 d after nematode inoculation, the chemical treatments were applied in each trial with the total number of treatments varying among trials. For the spirotetramat trial, there were four treatments including: (i) spirotetramat at 0.017 kg a.i./ha; (ii) spirotetramat at 0.026 kg a.i./ha; (iii) methylated seed oil blend (Drexel – MES-100) at 2.6 ml/L as an adjuvant control; and (iv) an untreated control. Foliar application rates were based on a spray coverage of spirotetramat at 76.6 L/ha. In the fluensulfone trial, there were only two treatments: (i) fluensulfone at 3.92 kg a.i./ha; and (ii) an untreated control. The fluensulfone rate 3.92 kg a.i./ha was converted from a volumetric rate of 4 mg a.i./L (4 ppm) of soil. For the spirotetramat trial, the low and high rate of spirotetramat, 0.92 ml/L and 1.4 ml/L, respectively, was separately mixed with MES-100 (2.6 ml/L solution) in 1,000 ml of tap water and applied till runoff as a foliar application to each plant using a spray bottle, as an adjuvant control treatment. Similarly, a 1,000 ml solution of MES-100 was applied as a foliar application to each plant, until runoff. In the fluensulfone trial, 14 mg of a.i. of fluensulfone was mixed in 200 ml of tap water and drench applied in four holes (10-cm-deep) made in the soil surface around each plant in each pot. Plants were not watered for a few days after drenching. Treated pots of each of the spirotetramat and fluensulfone trials were arranged in a randomized complete block design with six replications in the same greenhouse and conditions listed above. All plants were watered and fertilized with 1 ml of 13-13-13 fertilizer as needed until termination of trials.

The final population (pf) density of *M. incognita* in the soil and reproduction factor (Rf) were assessed at 40 and 70 d after inoculation (DAI) for both trials. Both trials were terminated 70 DAI and dry weights of both shoots and roots were recorded. To assess soil nematode population density in each pot, four soil cores (2.5-cm-diam. × 15-cm-deep) were collected randomly, 40 and 70 DAI from the area around each plant, and a composite soil sample was prepared. *M. incognita* J2 were extracted from a 100-cm^3^ soil sub-sample using soil sieves and centrifugal-flotation technique (Jenkins, 1964) and counted using a stereomicroscope. Also, the entire root system was washed free of soil and *M. incognita* eggs were collected from all the roots using a 0.5% NaOCl solution (Hussey and Barker, 1973). Then, the total population of *M. incognita* in each pot was determined by combining total numbers of J2 in soil and eggs extracted from the respective root system. Furthermore, the nematode reproduction factor (Rf = Pf/Pi) for both trials was also calculated by dividing total numbers of nematodes per pot (Pf = final population) by the number of nematodes added (Pi = initial inoculum) (Roberts and May, 1986). The spirotetramat and fluensulfone trials on *M. incognita* were repeated in 2012 under similar greenhouse conditions with the same treatments and statistical design, except that there were eight replications per treatment instead of six replications.

### Effect of a single application of spirotetramat and fluensulfone on the population density of *M. xenoplax*

In 2012, a third trial was conducted to compare the effects of both spirotetramat and fluensulfone against *M. xenoplax* as single foliar and drench applications, respectively. This trial was conducted under similar greenhouse conditions with the same treatments, statistical design, and replications as described for both spirotetramat and fluensulfone trials on *M. incognita*, the only exception being that the efficacy of both compounds were directly compared to each other in a single trial. For this trial, seedlings of ‘Nemaguard’ rootstock, planted as a susceptible host, were planted and prepared as described previously. After establishment, 1,000 mixed stages of *M. xenoplax* were used as inoculum, and the nematode population density in the soil and reproduction factor were assessed at 30, 60, and 90 DAI. This trial was repeated once as described above, except there were seven replications per treatment instead of six replications.

### Effect of dual application of spirotetramat on population densities of *M. incognita* and *M. xenoplax*

Two separate trials using a dual application of spirotetramat were also conducted with *M. incognita* and *M. xenoplax*. Due to restrictions to peach seedling growth within the greenhouse, only two applications of spirotetramat were evaluated against both nematodes. Protocols were similar to the previous single application trials expect that the treatments were applied twice, the first spirotetramat and (MES-100) adjuvant application occurring 10 DAI followed by a second application at 40 DAI. The treatments for both *M. incognita* and *M. xenoplax* trials included: (i) two applications of spirotetramat at each rate of 0.017 and 0.026 kg a.i./h, (ii) an untreated control, and (iii) two applications of MES-100 as an adjuvant control at 2.6 ml/L. The *M. incognita* trial was terminated 70 DAI, whereas the *M. xenoplax* trial was terminated at 90 DAI. The observations on nematode density and reproduction factors of *M. incognita* were recorded at 40 and 70 DAI, and of *M. xenoplax* at 30, 60, and 90 DAI. These trials were repeated once with similar greenhouse conditions and methods in 2012.

### Statistical analysis

All statistical analyses were performed using a generalized mixed model (GLIMMIX PROC, SAS Institute, Cary NC) to evaluate interactions between trials and if no significant interaction was detected, data were combined for analysis. Nematode and egg counts for each treatment were transformed using log_10_(*x* + 1). Means were separated by Fisher’s *t*-test using LSD *α* = 0.05.

## Results

### In vitro assays

In the first *M. xenoplax* assay, both rates of spirotetramat suppressed mobility compared to the untreated control after 24, 48, and 72 hr of exposure. Fluensulfone significantly decreased *M. xenoplax* mobility to a lower level than both rates of spirotetramat and the untreated control after 24, 48, and 72 hr of exposure as well (Table 1). Similar results were observed in the second *M. xenoplax* in vitro assay, with both rates of spirotetramat and fluensulfone significantly suppressing nematode mobility compared to the control after 24, 48, and 72 hr of exposure. The higher rate of spirotetramat provided greater suppression than the lower rate at 24 hr, but the effect was lost at 48 and 72 hr (Table 1).

**Table 1:** In vitro assay for the comparison of spirotetramat and fluensulfone on the mobility of *Meloidogyne incognita* and *Mesocriconema xenoplax*.

	*M. xenoplax*						*M. incognita*					
	% Motile nematodes^a^						% Motile J2					
	Assay 1			Assay 2			Assay 1			Assay 2		
Treatment	24 hrs	48 hrs	72 hrs	24 hrs	48 hrs	72 hrs	24 hrs	48 hrs	72 hrs	24 hrs	48 hrs	72 hrs
Untreated control	52.9 a^b^	48.9 a	60.1 a	56.9 a	44.8 a	56.2 a	94.0 a	90.9 a	94.7 a	98.8 a	99.3 a	99.5 a
Spirotetramat at 0.017 kg a.i./ha	36.3 b	32.1 b	43.3 b	47.8 b	30.5 b	35.3 b	94.8 a	94.0 a	96.5 a	98.1 a	98.5 a	99.7 a
Spirotetramat at 0.026 kg a.i./ha	33.2 b	24.7 b	34.0 b	32.4 c	32.1 b	32.4 b	93.0 a	93.9 a	91.6 a	98.7 a	98.4 a	99.7 a
Fluensulfone at 3.92 kg a.i./ha	17.1 c	8.05 c	4.79 c	28.9 c	25.5 b	19.2 c	48.2 b	46.2 b	11.0 b	65.6 b	8.56 b	2.23 b

**Notes:** Data are means of 12 replications (6 replications from the original assay + 6 replications from the repeated assay). ^a^% motile nematodes per 500 μL of solution. ^b^Means within a column followed by the same letter are not significantly different according to Fisher t-test using LSD *α* = 0.05.

For the first and second *M. incognita* assays, neither rate of spirotetramat was found to be effective in significantly reducing J2 mobility at the 24, 48, and 72 hr sampling times compared to the control. In contrast, fluensulfone significantly reduced J2 mobility on all three sampling times compared to both spirotetramat treatments and the untreated control (Table 1).

### Effect of a single application of spirotetramat and fluensulfone on population density of *M. incognita* and *M. xenoplax*

For the *M. incognita* trials, a single application of spirotetramat at the lower rate reduced the J2 population and eggs of *M. incognita* J2 in soil and from roots, respectively, compared to the untreated control at 40 DAI, but not at 70 DAI. In contrast, when spirotetramat was applied once at the higher rate, it showed no detrimental effects on nematode population densities at either 40 or 70 DAI (Table 2). The adjuvant control was analogous to the untreated control with no distinction between *M. incognita* populations sampled (Table 2). Fluensulfone was found effective in lowering the *M. incognita* population compared to the untreated control at 40 DAI. However, like spirotetramat at the lower rate, the nematode suppressive effect of fluensulfone was lost at 70 DAI (Table 3). No significant differences were observed between treatments in Rf levels of *M. incognita*, calculated at the completion of the trials (Tables 2 and 3).

**Table 2:** Effect of spirotetramat, on population densities of *Meloidogyne incognita* infecting ‘Lovell’ peach under greenhouse conditions 40 and 70 days after inoculation (DAI).

	40 DAI		70 DAI		70 DAI		70 DAI	
Treatment	RKN/100 cm^3^ soil^a^		RKN/pot^b^		RKN/g dry root^c^		Rf^d^	
Untreated control	1,162	a^e^	70,250	a	13,147	a	3.44	a
Adjuvant as control	1,004	a	50,966	a	11,253	a	2.48	a
Spirotetramat at 0026 kg a.i./ha	665	ab	51,101	a	7,173	a	2.48	a
Spirotetramat at 0.017 kg a.i./ha	443	b	32,729	a	5,862	a	1.56	a

**Notes:** Data are means of 14 replications (6 replications from the original trial + 8 replications from the repeated trial). ^a^RKN/100 cm^3^ soil = number of root-knot nematode juveniles and eggs extracted from root and soil in 100 cm^3^; ^b^RKN/pot = total J2/pot and number of eggs per root system. ^c^Total RKN per plant divided by total dry root weight. ^d^Nematode reproduction factor (Rf = Pf/Pi), where Pf = the final population level and Pi = initial inoculum level. ^e^Means within a column followed by the same letter are not significantly different according to Fisher t-test using LSD *α* = 0.05. Mean separation is based on log_10_(x + 1) transformed data.

**Table 3:** Effect of fluensulfone, on population densities of *Meloidogyne incognita* infecting ‘Lovell’ peach under greenhouse conditions 40 and 70 days after inoculation (DAI).

	40 DAI		70 DAI		70 DAI		70 DAI	
Treatment	RKN/100 cm^3^ soil^a^		RKN/pot^b^		RKN/g dry root^c^		Rf^d^	
Untreated control	1,162	a^e^	139,697	a	20,253	a	3.44	a
Fluensulfone at (3.92 kg a.i./ha)	268	b	102,292	a	12,221	a	1.68	a

**Notes:** Data are means of 12 replications (6 replications from the original trial + 6 replications from the repeated trial). ^a^RKN/100 cm^3^ soil = number of *M. incognita* J2 per 100 cm^3^ soil combined with number of eggs extracted from root segments obtained from 100 cm^3^ soil subsample. ^b^RKN/pot = total J2/pot and number of eggs per root system. ^c^Total RKN per plant divided by total dry root weight. ^d^Nematode reproduction factor (Rf = Pf/Pi), where Pf = the final population level and Pi = initial inoculum level. ^e^Means within a column followed by the same letter are not significantly different according to Fisher t-test using LSD *α* = 0.05. Mean separation is based on log_10_(*x* + 1) transformed data.

For the *M. xenoplax* trials, both rates of spirotetramat, applied once, were ineffective in suppressing *M. xenoplax* population densities compared to the untreated control at 30, 60, and 90 DAI. In contrast, fluensulfone was effective in suppressing the *M. xenoplax* population as compared to the untreated control at 30, 60, and 90 DAI. Fluensulfone also had a significantly lower Rf levels of *M. xenoplax* compared to the untreated control (Table 4), however, no significant differences in Rf levels of *M. xenoplax* were observed for the spirotetramat treatments compared to the water control. No significant differences in plant growth parameters were observed among treatments for both *M. incognita* and *M. xenoplax* trials.

**Table 4:** Effect of fluensulfone and spirotetramat, on population densities of *Mesocriconema xenoplax* infecting ‘Nemaguard’ peach under greenhouse conditions 30, 60, and 90 days after inoculation (DAI).

	30 DAI		60 DAI		90 DAI		90 DAI	
Treatment	Nematodes/100 cm^3^ of soil^a^						Rf^b^	
Untreated control	75	a^c^	194	a	1,557	a	5.29	a
Adjuvant as control	137	a	138	a	1,127	a	3.57	b
Spirotetramat at 0.026 kg a.i./ha	85	a	198	a	1,071	a	3.83	ab
Spirotetramat at 0.017 kg a.i./ha	84	a	99	a	1,441	a	5.08	ab
Fluensulfone at 3.92 kg a.i./ha	12	b	11	b	65	b	0.22	c

**Notes:** Data are means of 13 replications (7 replications from the original trial + 6 replications from the repeated trial). ^a^Total ring nematode count, all life stages, per 100 cm^3^ soil. ^b^Nematode reproduction factor (Rf = Pf/Pi), where Pf = the final population level and Pi = initial inoculum level. ^c^Means within a column followed by the same letter are not significantly different according to Fisher t-test using LSD *α* = 0.05. Mean separation is based on log_10_(*x* + 1) transformed data.

### Effect of dual application of spirotetramat on reproduction of *M. incognita* and *M. xenoplax*

When spirotetramat was applied twice at the lower rate, *M. incognita* population densities were significantly reduced compared to the untreated control at 40 DAI (Table 5). However, as with the spirotetramat single application trials, this suppression was not detected in the second sampling at 70 DAI, and 30 d after the second application, as compared to the untreated control. Similarly, the dual application of spirotetramat at the higher rate significantly reduced the *M. incognita* population densities compared to the untreated control at 40 DAI but not at 70 DAI (Table 5). No differences were observed in Rf levels, calculated at the completion of the trials, of *M. incognita* among each treatment (Table 5).

**Table 5:** Effect of dual applications of spirotetramat, applied 10 and 40 days after inoculation (DAI), on population of *Meloidogyne incognita* infecting ‘Lovell’ peach under greenhouse conditions 40 and 70 days after inoculation (DAI).

	40 DAI		70 DAI		70 DAI		70 DAI	
Treatment	RKN/100 cm^3^ soil^a^		RKN/pot^b^		RKN/g dry root^c^		Rf^d^	
Untreated control	485	a^e^	12,869	a	1,672	a	0.37	a
Adjuvant as control	503	a	7,440	a	1,596	a	0.64	a
Spirotetramat at 0.026 kg a.i./ha	221	b	3,427	a	328	a	0.17	a
Spirotetramat at 0.017 kg a.i./ha	203	b	6,788	a	1,301	a	0.34	a

**Notes:** Data are means of 14 replications (6 replications from the original trial + 8 replications from the repeated trial). ^a^RKN/100 cm^3^ soil = number of *M. incognita* J2 per 100 cm^3^ soil combined with number of eggs extracted from root segments obtained from 100 cm^3^ soil subsample. ^b^RKN/pot = total J2/pot and number of eggs per root system. ^c^Total RKN per plant divided by total dry root weight. ^d^Nematode reproduction factor (Rf = Pf/Pi), where Pf = the final population level and Pi = initial inoculum level. ^e^Means within a column followed by the same letter are not significantly different according to Fisher t-test using LSD *α*=0.05. Mean separation is based on log_10_(*x* + 1) transformed data.

The dual application of spirotetramat at both rates showed no difference in *M. xenoplax* population densities or Rf levels when evaluated against the untreated control (data not shown). No differences in plant growth parameters were observed among treatments for both *M. incognita* and *M. xenoplax* trials.

## Discussion

With the spirotetramat single application trials, only the lower rate of spirotetramat significantly suppressed the *M. incognita* population at 40 DAI, but no difference was observed at 70 DAI. Since the spirotetramat had to be absorbed by the plant and translocated to the roots, it is possible that the higher rate, along with the included adjuvant, was phytotoxic and elicited a defense response from the plant, thus blocking further absorption (Klittich, 2014). For these trials, chemicals were applied to the plants 10 d after inoculation for both nematodes species. At this point in time, most of the viable *M. incognita* J2 should have entered the roots and been in contact with the chemical via the vascular system. Some of the *M. incognita* egg inoculum would not have hatched immediately, and therefore, could have entered the roots after the effectiveness of the product had dissipated. Spirotetramat has been shown to be active within the roots for two or more weeks (Bruck et al., 2009; Smiley et al., 2011, 2012). According to the *M. incognita* in vitro assays, spirotetramat appears to have no effect in terms of J2 mobility. This lack of response may indicate that the chemical needs to be ingested by the nematode and cannot easily move through the cuticle of the *M. incognita* juveniles. Results from a recent study by Vang et al. (2016) showed that spirotetramat at 30, 60, or 90 ppm had no effect on *M. incognita* egg hatch. At the same time, spirotetramat is known to reduce fecundity and fertility of the organism, but has not shown nematicidal activity (Bruck et al., 2009). Given this level of activity, some of the nematodes were still reproducing and re-infecting the host. This response along with non-synchronous hatching may explain the loss of treatment effects at the second generation sampling. Future research should examine these products in peach field trials to determine long-term effects.

In terms of *M. xenoplax*, exposure to spirotetramat would differ to that of *M. incognita*, since spirotetramat is foliar applied and vascularly translocated (Bruck et al., 2009). *M. xenoplax* is ectoparasitic and feeds on cortex root tissue and not the vascular column (Hussey et al., 1992). Therefore, if spirotetramat is limited to the vascular column of the root and does not readily pass through the pericycle into the root cortex where *M. xenoplax* feeds, the resulting suppression could potentially be minimized. The *M. xenoplax* in vitro assays showed both rates of spirotetramat reduced mobility at all times points. This result seems to contradict recent work where it was shown that spirotetramat at various concentrations caused an arrest in juvenile development, but had no lethal effects against *C. elegans* (Vang et al., 2016). More work will need to be done to examine the true effect of spirotetramat on nematode mobility.

Another trial was conducted to examine dual applications of spirotetramat at 10 and 40 DAI. However, the spirotetramat dual application only suppressed *M. incognita* at 40 DAI. A possible explanation could be the peach seedlings in these trials were affected by the time of year and external factors that could affect the transport of the chemical to the root. It is also just as likely the *M. incognita* inoculum was affected by these same factors which may explain the low Rf levels for each treatment. What remained unclear is why the second application at 40 DAI had no effect on *M. incognita* population densities when sampled 70 DAI. One possible explanation is the chemical has more of an effect on the juveniles and less so with the adults (Smiley et al., 2011, 2012; Vang et al., 2016).

Fluensulfone effectively reduced the *M. incognita* population densities at 40 DAI, but this reduction did not carry over to 70 DAI. Fluensulfone drenching results from the greenhouse trial is consistent with previous trials by Everich and Schiller (2009) showing a nematicidal effect and partial systemic activity in roots. Like with the spirotetramat trials, fluensulfone was applied at 10 DAI. Given the activity of fluensulfone against *M. incognita* J2 in the bioassay, the exact reason the effect diminished at 70 DAI is not known. On the other hand, the effect of fluensulfone on *M. xenoplax* was encouraging with long-lasting effect toward the 90 DAI sampling in this trial. Since *M. xenoplax* is an ectoparasitic nematode, it is always in contact with the soil rhizosphere. Within the rhizos-phere, water, nutrients, metabolites, and chemicals, like fluensulfone, accumulate (McNear, 2013). Thus, *M. xenoplax* would be in constant contact with fluensulfone until the chemical starts to degrade. This also confirms the nematicidal activity of fluensulfone in cortex tissues (Oka et al., 2009). Currently, the mode of action for fluensulfone is unknown, but it worked well in reducing *M. incognita* and *M. xenoplax* population levels in all trials and reduced mobility at all sampling times for each in vitro assays.

In summary, we showed the potential of the post-plant nematicides, spirotetramat, and fluensulfone, for use in the control of *M. incognita* and *M. xenoplax* in peach. Among the two nematicides tested, fluensulfone had more nematicidal effects against *M. incognita* and *M. xenoplax*, in particular it had a longer term effect against *M. xenoplax*. The addition of a second application of spirotetramat improved its effect against *M. incognita*. The peach industry in the southeastern US and other major commodities throughout the US have long been in need of a viable replacement for soil fumigants and/or additional option for control of plant-parasitic nematodes. This research is a promising step in the right direction in terms of providing producers with another practical management strategy. Future work will need to be conducted to better understand the application timing of spirotetramat and fluensulfone in the orchard and in a long-term peach production system.
